# Scientific Publications on Primary Biliary Cirrhosis from 2000 through 2010: An 11-Year Survey of the Literature

**DOI:** 10.1371/journal.pone.0035366

**Published:** 2012-04-11

**Authors:** Baodong Qin, Yan Liang, Zaixing Yang, Renqian Zhong

**Affiliations:** Department of Laboratory Diagnostics, Changzheng Hospital, Second Military Medical University, Shanghai, China; University of York, United Kingdom

## Abstract

**Background:**

Primary biliary cirrhosis (PBC) is a chronic liver disease characterized by intrahepatic bile-duct destruction, cholestasis, and fibrosis. It can lead to cirrhosis and eventually liver failure. PBC also shows some regional differences with respect to incidence and prevalence that are becoming more pronounced each year. Recently, researchers have paid more attention to PBC. To evaluate the development of PBC research during the past 11 years, we determined the quantity and quality of articles on this subject. We also compared the contributions of scientists from the US, UK, Japan, Italy, Germany, and China.

**Methods:**

The English-language papers covering PBC published in journals from 2000 through 2010 were retrieved from the PubMed database. We recorded the number of papers published each year, analyzed the publication type, and calculated the accumulated, average impact factors (IFs) and citations from every country. The quantity and quality of articles on PBC were compared by country. We also contrasted the level of PBC research in China and other countries.

**Results:**

The total number of articles did not significantly increase during the past 11 years. The number of articles from the US exceeded those from any other country; the publications from the US also had the highest IFs and the most citations. Four other countries showed complex trends with respect to the quantity and quality of articles about PBC.

**Conclusion:**

The researchers from the US have contributed the most to the development of PBC research. They currently represent the highest level of research. Some high-level studies, such as RCTs, meta-analyses, and in-depth basic studies should be launched. The gap between China and the advanced level is still enormous. Chinese investigators still have a long way to go.

## Introduction

### Background

Primary biliary cirrhosis (PBC) is an immune-mediated chronic liver disease characterized by intrahepatic bile-duct destruction, cholestasis, and fibrosis. It can lead to cirrhosis and eventually liver failure [1]. The disease primarily affects women over 40 years of age (female preponderance (9–10∶1), although younger patients have been diagnosed, including children [2]. According to reported data, a latitudinal geo-epidemiological pattern has been proposed to describe development of PBC [3]. The disease is most common in northern Europe and North America, especially in Scandinavia, Great Britain, and the Northern Midwest region of the US. It is rarest in Australia. It has been suggested that the incidence of PBC is increasing over time. For example, in Sheffield, UK, the rates rose from 5.8 to 20.5 cases per million between 1980 to 1999 [4], [5]. Antimitochondrial antibodies in the serum are a hallmark of PBC. Ursodeoxycholic acid (UDCA) is currently the only FDA-approved medical treatment for PBC. Liver transplantation is performed in patients with advanced or late-stage PBC. However, the limited number of donors has narrowed the application of liver transplantation, it has also posed large economic burdens on patients and government.

With the growing incidence of PBC and other emerging issues, the scientific communities of different countries have gradually attached more importance to this condition. Researchers attempt to understand the pathogenesis of PBC and to develop new diagnostic methods and remedies. However, the trends in the annual numbers of scientific publications and in the quality of research performed in different countries are not immediately clear. We analyzed English-language papers on PBC published in international journals over the past 11 years (2000.1.1–2010.12.31) and investigated the quantity and quality of articles from different regions.

## Methods

In this retrospective study, we evaluated all articles related to PBC indexed in PubMed over the past 11 years. Our search Mesh term was “liver cirrhosis, biliary,” and searched within a specific date range (2000.1.1 and 2010.12.31) and publication language (English only). The information within all selected articles was drawn out independently by two investigators (BD Qin and Y Liang), who surveyed the titles, authors, abstracts, publication types, and other details. Discrepancies were resolved by review of the full text. The research output from different countries was determined using the first author's institutional affiliations. Impact factors (IFs) from 2000 to 2010 were determined using Thomson Reuters in ISI's Journal Citation Reports (JCR) [6]. In addition, the number of citations of every article was calculated using the Web of Science of ISI Database.

First, the total annual number of articles and externally funded publications related to PBC were calculated. Second, the publication types of the articles were analyzed. These included basic research, clinical research, case reports, reviews, commentaries, and other types of articles. Third, the accumulated and average IFs, and citations of every article were calculated to permit comparison of the quality of the publications, which were mainly from the top five countries, ranked by the total number of articles produced during the past 11 years (See Flowchart S1).

### Statistical analyses

Statistical analyses were performed using SPSS 18.0 software (IBM, New York, US), and statistical results are given in Tables and Figures. Results mostly show the trends and scientific contributions of different countries. The differences of contributions (including quantity and quality) to PBC by different regions were detected using the Kruskal-Wallis test, and the means were compared using one-factor ANOVA. The trends with respect to number of articles were analyzed via curvilinear regression. *P* values of less than 0.05 were considered significant. These were determined using two-tailed tests.

## Results

### Total number of articles

**Figure 1:**
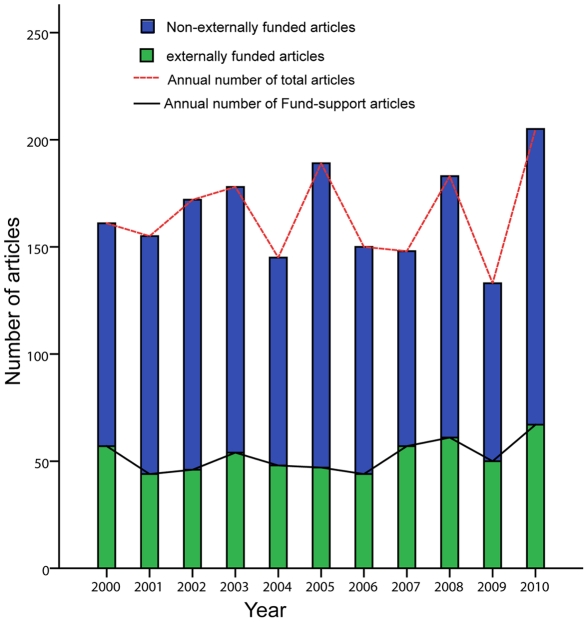
Number of articles published in journals worldwide during the past 11 years.

**Figure 2:**
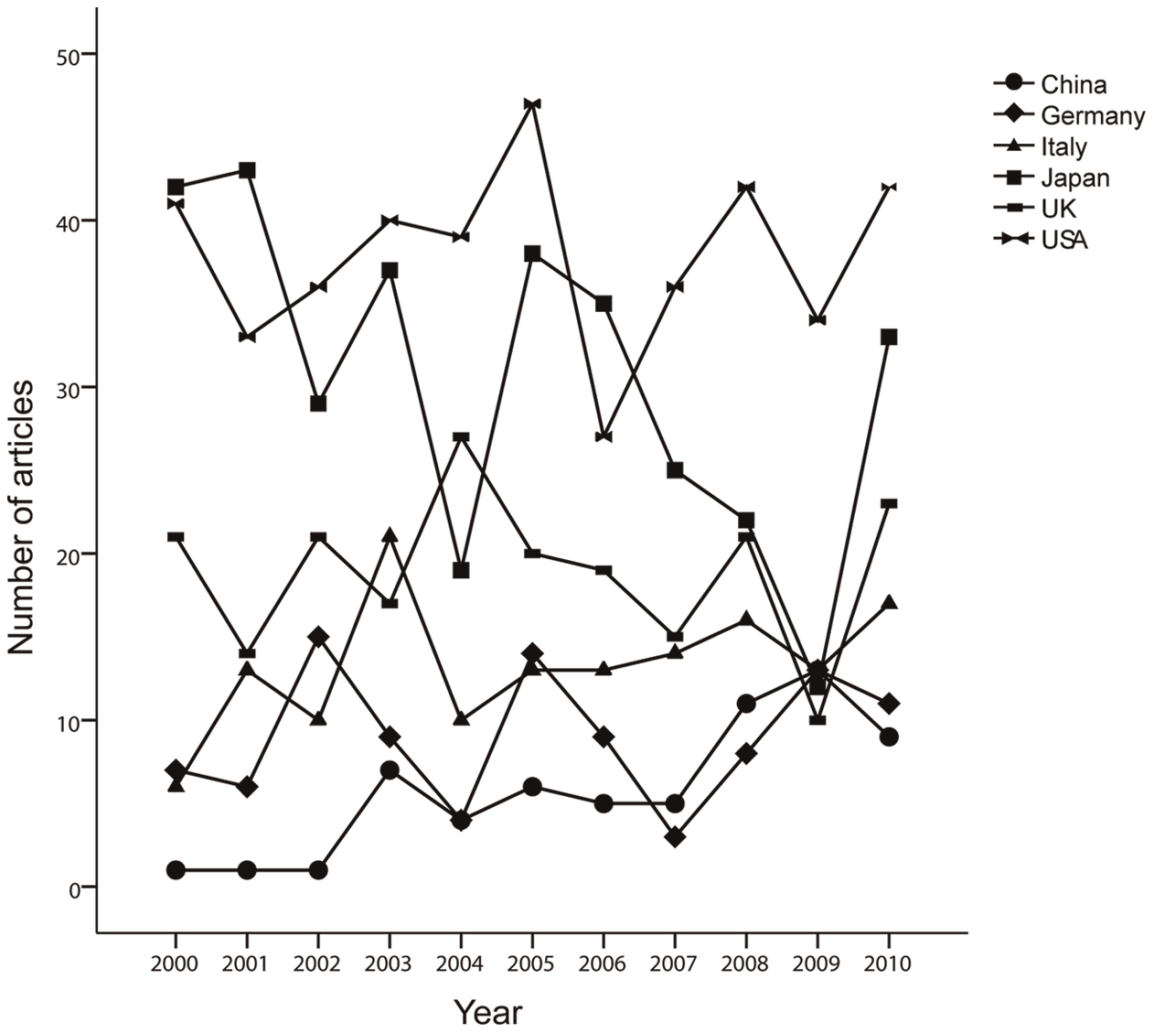
Trends in annual numbers of articles written by researchers from the six countries from 2000 to 2010.

From 2000 to 2010, a total of 1819 articles were published in the 389 journals indexed in PubMed. They were mainly from 52 countries. The number of articles published was largest in 2010 (205/1819) and least in 2009 (133/1819). However, the trend in annual number of articles published around the world fluctuated slightly and the number increased insignificantly from 2000 to 2010 (R^2^ = 0.023, *P* = 0.656). The number of externally funded articles exhibited the same trend as the total number of articles (R^2^ = 0.234, *P* = 0.131) (Fig. 1). The top five countries by number of articles were the US (22.9%, 417/1819), Japan (18.5%, 335/1819), UK (11.4%, 208/1819), Italy (8.0%, 145/1819), and Germany (5.4%, 99/1819). China (3.5%, 63/1819), including mainland China (35), Hong Kong (4), and Taiwan (24), ranked the seventh among the 52 countries. The growth in the annual number of published articles from these countries changed insignificantly, with the exception of China (R^2^ = 0.732, *P* = 0.001) (Fig. 2).

### Publication types

**Figure 3:**
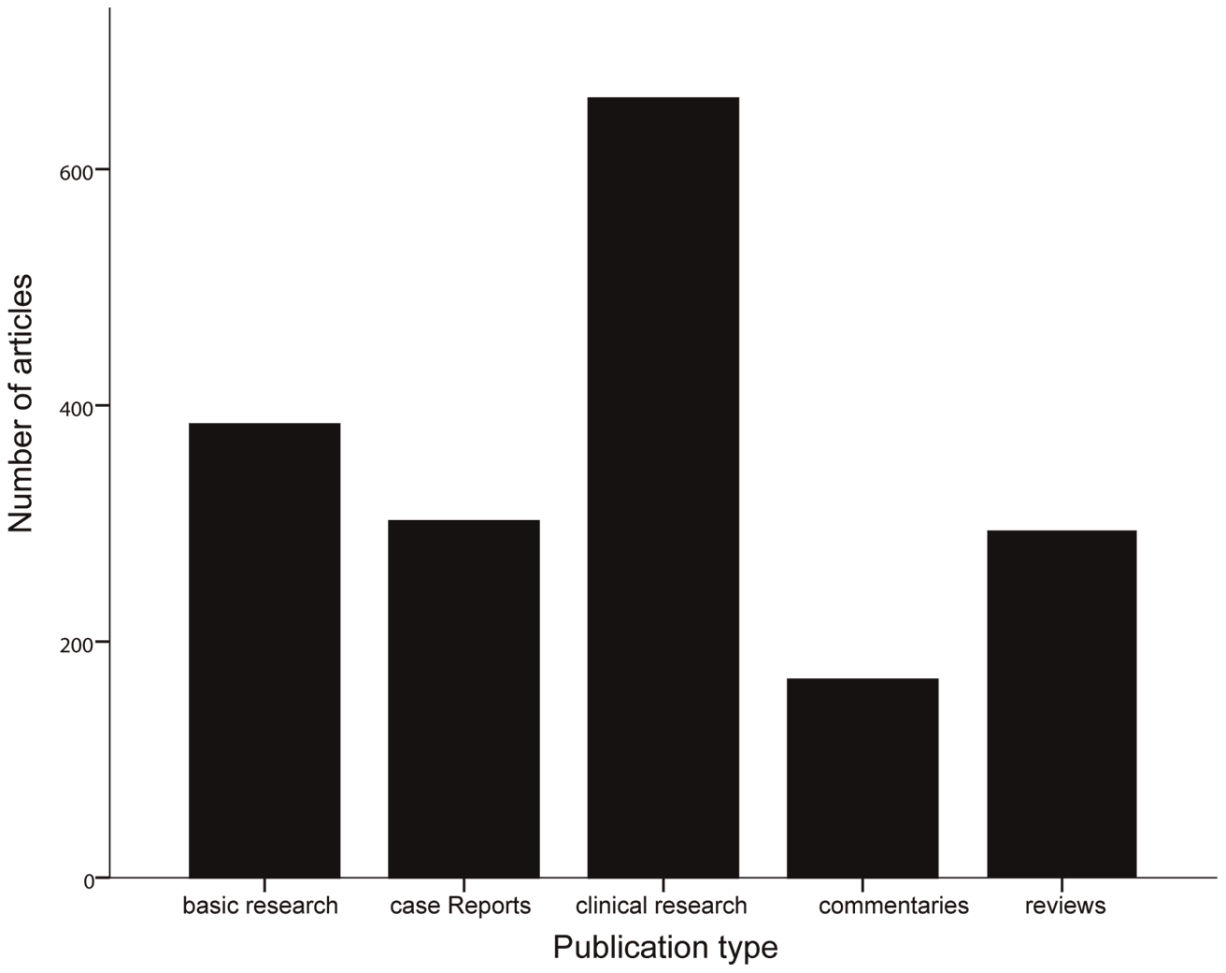
Total number of basic research, case reports, clinical research, commentaries, and reviews relating to PBC worldwide from 2000 to 2010.

**Figure 4:**
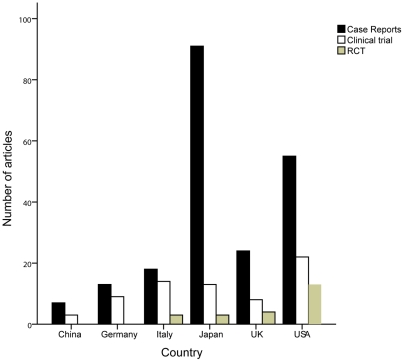
Number of clinical trials, RCTs, and case reports written by authors from the six countries between 2000 and 2010.

The number of published clinical studies was the largest, followed by basic studies, case reports, commentaries, and reviews (Fig. 3). Of all the 427 clinical studies, case-control studies occupied overwhelming share (66.98%), to which the authors from Japan, the US, and the UK made the main contributions. Japanese researchers published the most case reports, while US researchers conducted the most clinical trials (Fig. 4). However, there were only 38 RCTs in all 1819 articles. These were published by researchers from 11 countries. Although the number of RCTs was minor, there were still significant differences among the six countries (*P*<0.05), and the quantity of RCTs from the USA was found to be greater than those of other countries even though there were only 13 articles.

### Impact factors

**Figure 5:**
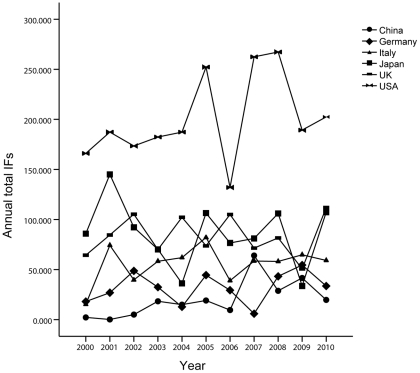
Annual total IFs of articles published by researchers from the top five countries and China from 2000 to 2010.

In accordance to the JCR, the 325 journals that published articles about PBC were indexed in SCI and had impact factors from 2000 to 2010, but 64 journals containing 91 articles had no impact factors. After excluding these 91 articles, we determined the impact factors of each one in its year of publication. Then we calculated the accumulated and average IFs of these countries. The annual total IFs from the US was significantly higher than those of other countries (*P*<0.05), and the four other countries showed complex trends and differed significantly despite the insignificant difference between the UK and Japan (*P* = 0.622)(Fig. 5). In addition, the average IFs were similar to above-mentioned results shown in Table 1, and the average IFs differed significantly among the six countries (*P*<0.05).

**Table 1:** Average impact factors (IF) of articles written by authors from six countries from 2000 to 2010, based on the annual IFs of the journals published.

Year	USA	UK	Canada	Germany	Italy	China
2000	4.576	4.907	2.204	3.688	4.484	1.586
2001	3.627	4.347	1.745	4.093	2.334	2.497
2002	4.457	4.727	5.173	3.704	1.180	0
2003	7.940	1.928	2.444	2.573	1.822	2.424
2004	3.042	3.398	4.249	1.934	2.426	0
2005	2.765	2.718	3.306	2.394	3.432	2.166
2006	5.567	6.463	5.991	5.717	3.771	1.795
2007	3.120	3.584	2.890	3.250	4.394	2.314
2008	5.139	3.830	3.983	4.385	3.006	3.480
2009	4.550	4.065	2.836	3.496	4.500	3.863
2010	5.134	4.120	5.625	5.106	4.966	2.899
Total	4.515	4.046	3.972	3.787	3.560	2.681

### Citations

**Figure 6:**
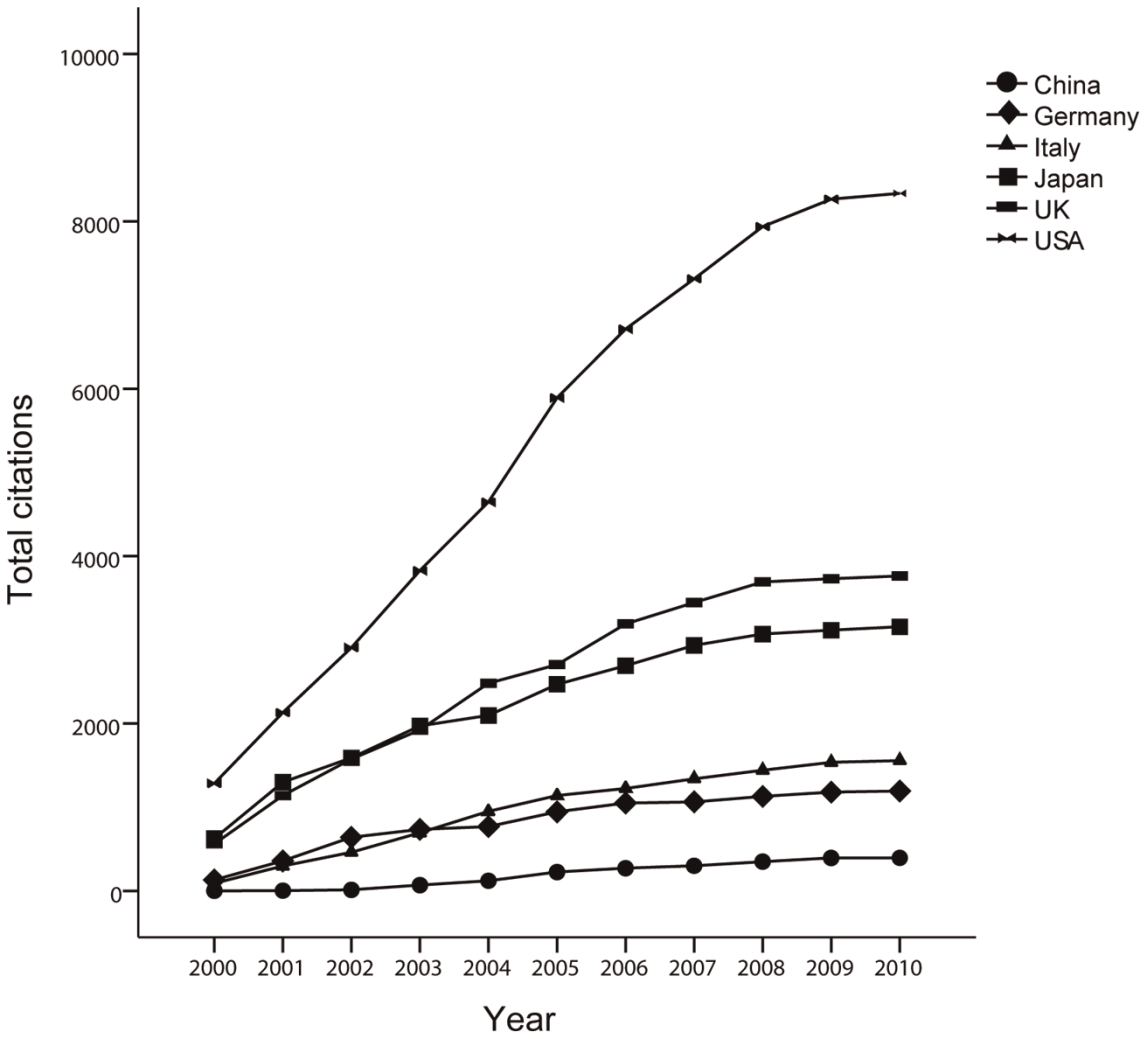
Annual citations of articles related to PBC written by authors from the top five countries and China from 2000 to 2010.

**Figure 7:**
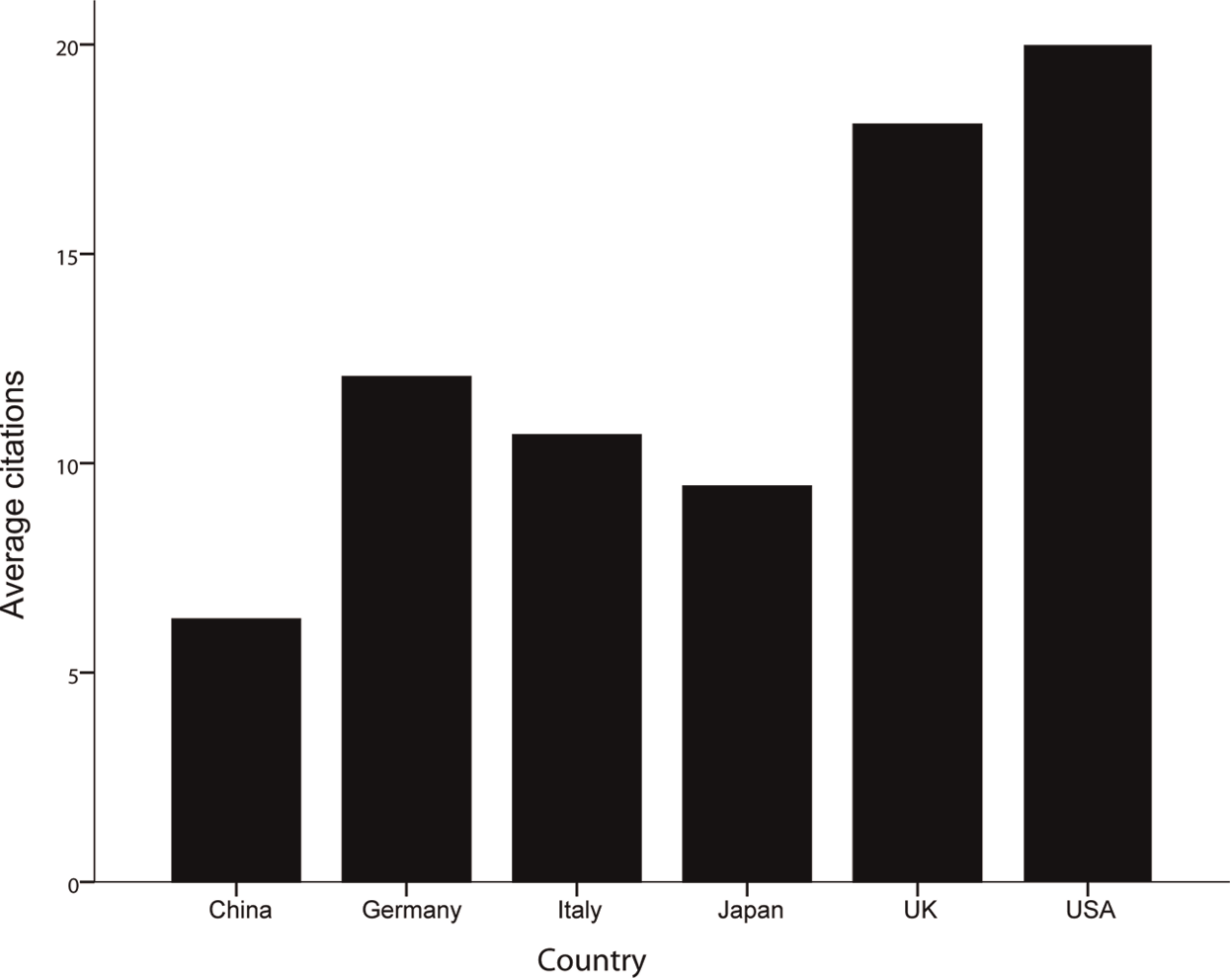
Average number of citations in articles published by researchers from the different countries during the past 11 years.

In the ISI database, we found the number of citations for every article and analyzed the total number of citations and the average citation rate from the top 5 countries and China. The results are shown in Figure 6. The US had 417 articles and 8334 citations. The UK had 208 articles and 3763citations, Japan had 335 articles and 3156 citations. Italy had 146 articles and 1558 citations. Germany had 99 articles and 1194 citations. China had 63 articles and 395 citations. These differences among these six countries were found to be significant (*P*<0.05). Papers from the US and UK had the greatest average number of citations (19.99 and 18.09), followed by Germany, Italy, and Japan (12.06, 10.67, and 9.45) (Fig. 7). Chinese papers had the fewer citations on average than papers from the above 5 countries.

### Favorite and most high-impact medical journals

The most popular journals are listed in Table 2. *Hepatology* was the No. 1 journal with respect to number of articles about PBC, followed by *J Hepatol*, *Am J Gastroenterol* and *Liver Int*. *Hepatology* also appeared among the top four journals of the five countries. A total of 416 articles from 2000 to 2010 were published in the top five ranking sub-specialty medical journals, which included *Hepatology, Gastroenterology, Gut, J Hepatol*, and *Am J Gastroenterol*. Only 19 articles on PBC were published in the four leading general-medicine journals: *NEJM, Lancet, JAMA*, and *BMJ*. There were only 3 articles published in the top three basic medical journals (*Nature*: 2 articles, *Science*: 1 article, *Cell*: 0 articles).

**Table 2:** The four most popular journals for authors from five countries between 2000 and 2010.

Rank	Total	USA	Japan	Germany	UK	Italy
1	Hepatology(86)	Hepatology(28)	Intern Med(24)	Hepatology(14)	Hepatology (10)	Dig Liver Dis(8)
2	Am J Gastroenterol (63)	Dig Dis Sci(24)	J Gastroenterol (21)	Dtsch Med Wochenschr (10)	Semin Liver Dis(8)	Am J Gastroenterol(7)
3	J Hepatol(60)	Am J Gastroenterol (21)	Nihon Shokakibyo Gakkai Zasshi (21)	Am J Gastroenterol (9)	Eur J Gastroenterol Hepaatol(7)	J Hepatol(5)
4	World J Gastroenterol (43)	Clin Liver Dis(13)	Nihon Rinsho (19)	J Hepatol(9) Z Gastroenterol (9)	Clin Liver Dis(6) J Hepatol (6)	Minerva Gastroenterol Dietol(5)

## Discussion

This is, to our knowledge, the first study to analyze the distribution of English-language papers on PBC from 2000 to 2010, and it is also the first report to evaluate the quantity and quality of the top five countries as ranked by number of articles.

The present study shows that the total number of articles about PBC has not varied drastically. Although that number reached the peak in 2010, the rate of increase is not considerable. The trend in the annual number of articles has not shown any evident increase, probably because the studies on PBC have reached a bottleneck or found some kind of limiting factor. In addition, a total of 575 articles (31.6%) on PBC were found to have been externally funded, and the quality of funded studies was much higher with respect to both average IFs and number of citations (*P*<0.05). This suggests that societies and governments should pour more funds into PBC research.

Clinical studies made up a considerable proportion of the articles evaluated, but highly convincing RCTs were rare. Only 17 meta-analyses were published during the past 11 years, and they mainly evaluated the effects of therapy rather than risk factors, diagnostic criteria, or prevention. Although basic studies made up the second largest subgroup, only 3 of these articles were published in the top three journals. Therefore, the scientific levels of these basic studies are probably not high and need to be improved. Case reports mainly discussed specific symptoms and complications. Japanese researchers contributed the most case reports (28.2%). Researchers should consider summarizing and classifying these findings.

These five developed countries, the US, UK, Japan, Italy, and Germany, have been in the forefront of global scientific research for many years, and authors from these countries have contributed the most to PBC with respect to both quantity and quality of published articles. The US clearly leads other countries in research productivity (accounting for 22.9% of the total), with an annual number of articles exceeding any of the other countries in the past 11 years, except in 2000, 2001, and 2006. Japan followed the US, but the annual number of papers from Japan has shown a downward trend, reaching its lowest point in 2009. None of the three other countries surpassed the US or Japan in number of articles except in 2004. The ranking with respect to number of articles was the UK, Italy, and Germany in the third, fourth, and fifth places. In addition to the number of articles, US researchers have also written the papers of the highest quality (*P*<0.05), with the highest annual total and average IFs. There were no significant differences in annual total IFs between Japan and the UK, but the average IFs of articles from Japan were significantly lower than those from the UK (*P*<0.05). Italians have published more top-quality articles than Germans with respect to both annual total and average IFs. In addition to these five countries, Canada and France also showed advanced-level PBC research. Researchers from these two countries contributed 4 articles in *NEJM* (France: 3 articles, Canada: 1 article) during the past 11 years.

Citations are another indicator of publication quality, showing the degree to which the paper has been accepted by other authors in the same field. Articles from the US exceeded those of all other countries with respect to average number of citations (*P*<0.05) during the past 11 years, and the cumulative number of citations is also fairly high. The UK surpassed Japan with respect to total number of citations in 2003, which was similar to the relationship between Germany and Italy. In 2010, the ranking with respect to total number of citations was US, UK, Japan, Italy, and Germany.

In the present study, *Hepatology* (163 articles) was found to be the most popular journal (8.9%) for authors writing about PBC. It printed 71 articles from the US, most of which were written by the researchers from the University of California at Davis School of Medicine (Gershwin ME). This laboratory represents the most advanced level of PBC research, where the researchers make a profound contribution to development of the PBC knowledge base. A total of 416 articles were published in the five highest-impact hepatology and gastroenterology journals, three of which are the top three popular journals with respect to total number of PBC articles. However, *Gut* and *Gastroenterology* published fewer articles (41 articles in *Gut*, 46 articles in *Gastroenterology*). For the four leading medical journals, only 19 articles were (*NEJM*: 11 articles, *Lancet*: 6 articles, *JAMA*: 1article, *BMJ*: 1article). It can be seen from the above results that the number of PBC articles published in top journals is still relatively low.

Although China is ranked No. 7 with respect to total number of articles on PBC during the past 11 years, the number has shown a significant upward trend (R^2^ = 0.732, *P* = 0.001). The total number of citations of publications from China are still below the internationally advanced level, such as that observed in the five countries discussed in present study. However, the annual total IFs of articles has also increased each year, reaching a peak in 2007. The average IFs from China (3.651) surpassed Japan (2.971) and Germany (3.532), but it not to a statistically significant extent (*P* = 0.514 and *P* = 0.908). From the analysis of publication types, none of the RCT articles were written by researchers from China. With the increases in the number of PBC patients, Chinese researchers should launch more multicenter prospective studies and RCTs aimed at risk factors, diagnosis, complications, treatments, and prevention. In conclusion, the contribution of China to PBC research is less than that of the top five countries. Therefore, there is a lot of room for Chinese researchers to improve.

Actually, there may be some limitations in the literature. First, the six countries selected may not represent the status of all articles and may also not be representative of the international level of PBC research. However, these countries have produced 70% of the total number of English-language articles published during the past 11 years, which suggests that research in these countries is advanced. Second, we only retrieved English-language documents and passed over articles published in other languages. Although English is the international language of communication in academic fields and high-quality articles and journals are mainly published in English, there are still 290 (13.7%) articles related to PBC indexed in the PubMed database published in non-English languages during the past 11 years. These include 37 French papers, 48 German papers, 46 Japanese papers, 58 Chinese papers, 30 Spanish papers, 8 Italian papers and more. This may have affected the efficacy and accuracy of the current assessment of the development of PBC research around the world because some researchers may release articles on PBC in their own languages rather than in English. Third, it may not be appropriate or accurate to assess the quality of these articles using IFs [7]. There are many inherent limitations to IFs as quantitative measurement tools. Only when citations are uniformly spread between articles can we use IFs to objectively evaluate the quality of individual articles. However, the non-parametric distribution of citations to articles lies at the heart of the problem with impact factors. Some journals include many useful articles, accounting for most of the total number of citations of journals [8]. Moreover, document type can affect IF. Review papers attract more citations than original research papers [9]. Original research papers, however, are the main engines that generate new knowledge, and so are the best representatives of research levels. In the present study, increases in the IFs of articles related to PBC may paradoxically reduce the overall influence of said research on the scientific community because fewer original research papers will be published. For the reasons given above, other metrics that assess the quality and quantity of citations have been proposed, such as the Eigenfactor Score and Article Influence Score, which must be taken into account when attempting to determine impact factor [10]. However, Bollen et al. found that scientific impact is a multi-dimensional characteristic that cannot be adequately captured by one metric [11]. Any single indicator may not accurately indicate the quality and impact of articles or academic journals. Despite its many at present unavoidable limitations, the journal impact factor is still the most commonly used indicator of the quality or at least the popularity of a given scientific journal.

In conclusion, the present study provides some useful information regarding scientific studies of primary biliary cirrhosis. US researchers have contributed the most to the development of PBC studies, and the country currently produces the highest level of PBC research. More RCTs, meta-analyses, and high-level basic studies should be carried out. Although the quantity and quality of articles written by Chinese scientists have increased every year, the gap between China and the advanced level in PBC research is still enormous.

## Supporting Information

Flowchart S1 The main flowchart of this study about scientific publications on primary biliary cirrhosis.
(DOC)
