# Supplementary material for: Scientific Publications on Primary Biliary Cirrhosis from 2000 through 2010: An 11-Year Survey of the Literature
Source: PLoS One. 2012 Apr 11;7(4):e35366. doi: 10.1371/journal.pone.0035366 (PMC3324556; doi:10.1371/journal.pone.0035366)
Supplement: Flowchart S1 — The main flowchart of this study about scientific publications on primary biliary cirrhosis. (DOC) [file pone.0035366.s001.doc]

**Flowchart S1:**

| Search in PUBMED database  Search Mesh: ”liver cirrhosis, biliary” |
| --- |

| Inclusion criteria:  Publication language: English  Time limited :2000 to 2010 |
| --- |

| The total number of articles: 1819  Choose the top five countries in number of articles related to PBC and China |
| --- |

| \| USA  419 articles \| Japan  335 articles \| UK  208 articles \| Italy  145 articles \| Germany  99 articles \| China  63 articles \| \| --- \| --- \| --- \| --- \| --- \| --- \| |
| --- | --- | --- | --- | --- | --- | --- |

| Search the Impact factor and Citations of every article from the above-mentioned countries, besides the publication type or popular journals |
| --- |

| Evaluated the quantity and quality of the articles form these six countries |
| --- |

| Discussed the contribution to PBC research from these counties and the gap between China and others |
| --- |
